# Detection of Aberrant Carotid Arteries Prior to an Adenoidectomy: A Case Report

**DOI:** 10.7759/cureus.73042

**Published:** 2024-11-05

**Authors:** Shiven Sharma, Ronit Sethi, Tony Han, Nathan Ji, Michele M Carr

**Affiliations:** 1 Otolaryngology, Icahn School of Medicine at Mount Sinai, New York, USA; 2 Otolaryngology, University at Buffalo Jacobs School of Medicine and Biomedical Sciences, Buffalo, USA

**Keywords:** aberrant internal carotid artery, adenoidectomy, pediatric otolaryngology, preoperative imaging, surgical risk factors

## Abstract

Aberrant carotid arteries are rare vascular anomalies that can significantly complicate head and neck surgeries, particularly in pediatric patients. These anomalies may be asymptomatic and are often discovered incidentally on imaging studies performed for unrelated conditions. The failure to recognize these anomalies preoperatively can result in life-threatening complications, such as catastrophic hemorrhage. This case report highlights the critical importance of thorough imaging and cautious surgical planning in preventing such outcomes.

A female pediatric patient presented with nasal obstruction, recurrent ear infections, and otorrhea. A lateral soft tissue X-ray was performed to assess adenoid size and revealed an unexpected retropharyngeal fullness, initially raising concerns for a retropharyngeal abscess. A subsequent CT scan identified medialized aberrant carotid arteries lying in the posterior oropharynx, posing a significant risk for surgical intervention. In accordance with these findings, the patient underwent adenoidectomy with careful intraoperative assessment and planned contingencies. The patient remained asymptomatic with no vascularity, such as bulging or pulsation, on the posterior pharyngeal wall. Her adenoid was successfully removed, and she recovered without complications.

This case underscores the importance of preoperative imaging and vigilance in identifying vascular anomalies that can complicate surgery. The absence of intraoperative vascular signs highlights the potential for occult anomalies to go undetected. Surgeons should consider the possibility of such anomalies when encountering unusual imaging findings.

Recognizing aberrant carotid arteries is crucial in preventing catastrophic complications during head and neck surgeries. Preoperative imaging and planning play a vital role in identifying these risks and are essential in ensuring patient safety.

## Introduction

Aberrant carotid arteries are anatomic vascular anomalies that can significantly complicate head and neck surgeries, particularly in pediatric patients. The Weibel and Fields classification system (1965) has traditionally described tortuosity, coiling, and kinking as the three types of anatomic internal carotid artery (ICA) aberrances [1], although recent studies have increasingly considered factors like the ICA-to-pharyngeal wall distance and the pharyngeal level in assessing otolaryngological surgery risk [2]. Tortuosity involves any C- or S-shaped elongation of the ICA; coiling refers to elongation of the ICA causing a circular loop; and kinking involves an abnormal angling of the ICA associated with stenosis [1]. Under the Weibel-Fields system, a ~5-6% incidence of high-grade ICA aberrance has been estimated among the general population, making ICA abnormality more common than originally believed [3].

Because ICA aberrances are asymptomatic in >90% of cases, they are rarely recognized pre-operatively, and often discovered incidentally either on imaging studies for unrelated conditions or intra-operatively [4]. The failure to recognize carotid artery anomalies, however, can cause catastrophic complications in otherwise routine surgeries like adenoidectomies or tonsillectomies, especially among children who have higher variance in surgical risk parameters like the ICA-to-pharyngeal wall distance [5]. Life-threatening post-operative hemorrhage in these procedures is extremely low, but cases of surgical ICA dissection have been described and analyzed in the literature [6-8]. Meanwhile, for tonsillectomies, hemorrhage still remains the most prevalent life-threatening complication reported [9]. This case report will highlight the presence of an ICA abnormality in a one-year-old female patient undergoing adenoidectomy and, in turn, demonstrate the importance of keeping this variation in mind whenever an adenoidectomy is undertaken.

## Case presentation

**Figure 1:**
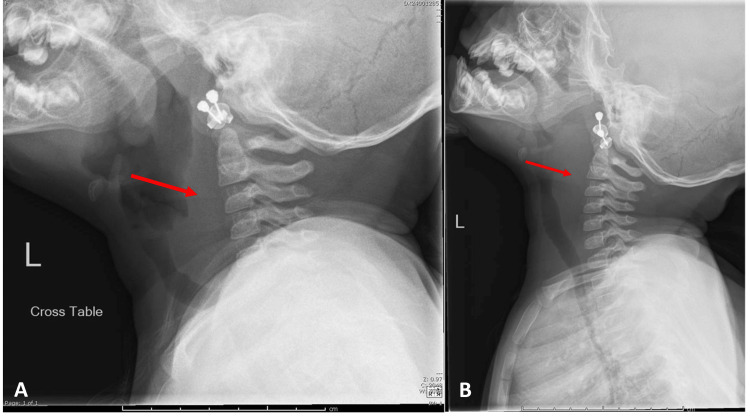
Soft tissue lateral X-rays demonstrating retropharyngeal widening (red arrows)

A one-year-old female presented with nasal obstruction, recurrent ear infections, and otorrhea. Her symptoms began around nine months of age when recurrent ear infections and nasal congestion were first noted at a pediatrician's visit. At approximately 10 months, she underwent bilateral tympanostomy tube placement, after which recurrent otorrhea developed within a week. She had no history of epistaxis, mouth breathing, or snoring, and she slept well at night, thus showing no signs that would indicate a need for a sleep study or concerns for obstructive sleep apnea. The initial evaluation included a lateral soft tissue X-ray to assess adenoid size (Figure 1). The X-ray showed a large adenoid but also revealed an unexpected retropharyngeal fullness, initially raising concerns for a retropharyngeal abscess. However, there are multiple possibilities when considering a differential diagnosis as outlined in Table 1. A repeat soft tissue lateral X-ray ordered by the radiologist still showed a widened retropharynx.

**Table 1:** Differential diagnoses for retropharyngeal fullness/widening on X-rays Adapted from [10-12].

Cause	Description	Clinical features/considerations
Retropharyngeal abscess	A collection of pus in the retropharyngeal space, often due to infection (e.g., upper respiratory or dental infections).	Fever, neck stiffness, difficulty swallowing (dysphagia), drooling, respiratory distress, and history of infection.
Congenital vascular anomaly	Anomalous positioning of vascular structures (e.g., medialized internal carotid artery) that can appear as retropharyngeal fullness.	Typically asymptomatic; may be found incidentally during imaging for other concerns.
Trauma	Injury to the cervical spine or soft tissues leading to hemorrhage or edema in the retropharyngeal space.	History of trauma, neck pain, and neurological symptoms if spinal cord involved.
Neoplasm	Tumors (benign or malignant) in the retropharyngeal space or surrounding structures (e.g., sarcomas, lymphomas).	Gradual onset of symptoms like dysphagia, hoarseness, weight loss, and neck mass.
Spondylodiscitis	Inflammation of the vertebrae and intervertebral discs, often secondary to infection (e.g., tuberculosis, osteomyelitis).	Fever, neck pain, neurological symptoms if spinal cord compression is present.
Adenoidal hypertrophy	Enlargement of the adenoids, common in children, which may cause widening of the retropharyngeal space.	Chronic nasal congestion, snoring, mouth breathing, and recurrent ear infections.
Other infections	Pharyngitis, tonsillitis, or cellulitis can cause reactive lymphadenopathy and soft tissue swelling.	Sore throat, fever, lymphadenopathy, and general malaise.

Diagnostic workup

**Figure 2:**
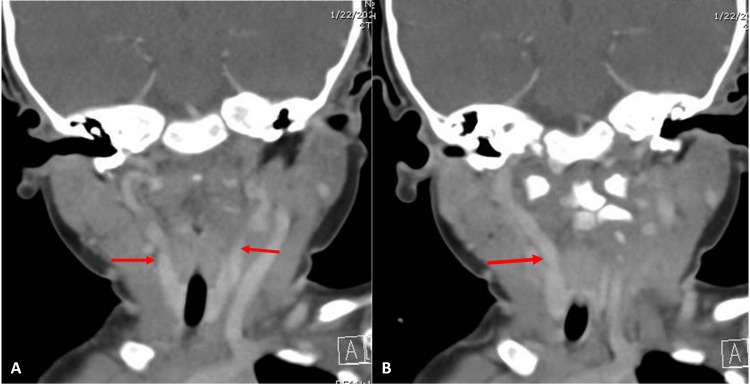
CT scan demonstrating medialized carotid arteries (red arrows) Note that there was likely movement artifact as the CT scan was obtained without sedation, and there was likely movement.

To further investigate the retropharyngeal fullness, a CT scan with contrast material was performed (Figure 2). The CT scan identified medialized aberrant carotid arteries lying in the posterior oropharynx, posing a significant risk for surgical intervention.

Surgical planning and intervention

Given the critical findings from the imaging studies, counseling of her parents was done and the risks and benefits of adenoidectomy were reviewed. Her parents elected to proceed with surgery. A cautious surgical approach was adopted with a plan to do a partial adenoidectomy if the vessels were clinically apparent; if there were pulsations underlying the adenoid, the procedure would be aborted. The expectation was that the vessels were inferior to the adenoid, and the surgery would likely be safe. The patient underwent a coblator adenoidectomy with careful intraoperative assessment and planned contingencies. There were no curettes or other cutting instruments used. During the intraoperative examination, the patient did not display any abnormality of the posterior nasopharyngeal or pharyngeal regions, such as bulging or pulsation.

Outcome

The adenoid was successfully removed, and the patient recovered without complications.

## Discussion

Although ICA aberrations rarely lead to surgical complications in practice, they are a significant risk factor for severe debilitative outcomes or death among pharyngeal surgeries [13]. Mortality after tonsillectomy has been described in one in 1000 to 170,000 patients [14]. Moreover, nearly 30% of deaths are mainly related to the hemorrhage secondary to the lesion of the ICA and its branches [14]. High-grade ICA aberrance has a 5-6% incidence, while moderate-grade has an estimated 12-26% incidence, making ICA variation relatively common among the general population [2].

Typically, the ICA consists of seven anatomic segments, with the lower bound cervical segment (C1) following a relatively straight ascending course from the common carotid artery bifurcation point, through the carotid sheath, into the carotid canal of the petrous bone (C2) [15]. Subsequent segments of the ICA turn several times at the skull base before bifurcating into the posterior communicating and anterior choroidal arteries [15]. Individuals with aberrant, surgically risk-prone ICAs have tortuosity, coils, or kinks in this C1 ICA segment, generating issues like redundancy and decreased ICA-pharyngeal wall distance [1]. This can occur at the nasopharyngeal, oropharyngeal, or hypopharyngeal levels [16]. For pediatric populations, this is an especially relevant concern. The ICA-to-pharyngeal wall distance increases exponentially from 14 mm in a one-year-old to a maximum of 25 mm in 12-15 year-olds [5]. Young children, for whom adenoidectomies and tonsillectomies are most common, have an already low and more variable ICA-pharyngeal wall distance, translating to a heightened risk for ICA dissection and uncontrollable bleeding in the case of unidentified aberrance. Although it is standard practice to examine for pulsations in the pharyngeal wall to rule in a medialized carotid artery, sometimes pulsations cannot be detected due to low arterial pressure under anesthesia or because of the type of aberrance [16].

The underlying cause of ICA aberrance is still not fully clear. Theories around age-driven vascular degeneration and congenital development have been previously proposed. However, based on several lines of evidence, including a frequent symmetric, bilateral incidence of ICA aberration and the presence of ICA aberration at relatively equal rates between young children and adults [1,12], it seems likely that severe ICA aberration is driven congenitally but negatively modified by vascular degeneration and elasticity loss [17]. To this effect, it has been postulated that fetal development issues with unlooping of the ICA about its origin points at the third branchial arch artery and cranial portion of the dorsal aorta are the etiological roots [2]. In theory, this could lead to the retention of looping or kinking in the vessel that carries into adulthood. At present, there is no evidence to demonstrate that this is the case definitively.

Compounding the issue of surgical risk from ICA aberrance is the frequent lack of symptomology in both children and adults. Among the <10% of patients with symptoms potentially related to ICA anomaly, dysphagia, hoarseness, foreign body sensation, and upper respiratory issues are most common [18]. These symptoms, however, rarely present in children. Studies in the neurology literature have hypothesized associations between ICA aberrance and neurologic disorders (e.g., hemiplegia) due to blood flow alteration and cerebrovascular insufficiency, particularly in kinked ICAs [19,20]. This notion is still heavily debated in the literature due to the lack of studies showing causation.

In common pharyngeal surgeries like adenoidectomies and tonsillectomies for children, preoperative imaging is rarely conducted. Presumably, this is to help minimize costs, resource usage, patient time, and radiation exposure, especially given the low incidence of severe ICA aberration. Alarmingly, a study of pediatric pharyngeal surgery patients found that 66% of ICA anomalies were discovered intraoperatively as opposed to preoperatively [16]. This can pose a serious risk due to limited time for surgical replanning, especially when aberrance is discovered mid-excision. For this reason, it is essential that otolaryngologists are aware of the possibility of ICA aberrance and examine meticulously for pharyngeal pulsations prior to incision, thereby decreasing reliance on imaging in order to minimize radiation exposure. In the case where aberrance is suspected at all, the use of preoperative or intraoperative Doppler ultrasonography has been recommended to investigate ICA aberrance type and assess risk potential, ultimately aiding in decision-making [4]. Risk gradation could be done, for example, using the clinicoradiologic grading system proposed by Pfeffer et al., which accounts for the ICA-to-pharyngeal wall distance and the pharyngeal level [4]. However, it is ultimately dependent on the otolaryngologist’s discretion and surgical confidence as to whether or not to proceed with the surgery.

## Conclusions

This case report underscores the importance of vigilance in identifying vascular anomalies that can complicate surgery. Preoperative imaging is not always available prior to adenoidectomies, but when it is, images should be evaluated carefully. Recognizing aberrant carotid arteries is crucial for minimizing the risk of catastrophic surgical complications and ensuring patient safety. The absence of intraoperative vascular signs highlights the potential for occult anomalies to go undetected. Surgeons should consider the possibility of such anomalies when encountering unusual imaging findings.
